# Cutaneous angiosarcoma in a super-morbidly obese patient (body mass index 106.7 kg/m^2^) highlighting diagnostic and therapeutic barriers in extreme obesity: a case report

**DOI:** 10.1186/s13256-026-06391-3

**Published:** 2026-07-24

**Authors:** Maria Fueth, Christoph Wallner, Yonca Steubing, Tom Alexander Huyghebaert, Alexander Wolff, Simon Bausen, Marcus Lehnhardt, Felix Reinkemeier

**Affiliations:** Department for Plastic and Hand Surgery, BG University Hospital Bergmannsheil Bochum, BG University Hospital Bergmannsheil, Ruhr University Bochum, Bürkle-de-La-Camp Platz 1, 44789 Bochum, Germany

**Keywords:** Extreme obesity, Lymphangiosarcoma, Super-obesity, Lymphedema, Public health, Angiosarcoma, Health service accessibility

## Abstract

**Background:**

With the rising prevalence of extreme obesity, healthcare systems are increasingly confronted with patients who exceed the physical limits of standard diagnostic and therapeutic infrastructure. When body habitus exceeds the physical limitations of imaging and procedural infrastructure, timely cancer staging and intervention may become impossible, directly impacting survival.

**Case presentation:**

We report a 54-year-old White woman (weight ≈ 280 kg, BMI 106.7 kg/m^2^) with chronic stage IV lymphedema of both lower limbs who presented with a rapidly enlarging, violaceous, ulcerated plaque on the left calf. Histopathology confirmed high-grade cutaneous angiosarcoma (Stewart–Treves syndrome). Standard staging with CT, MRI, and positron emission tomography (PET) was not feasible due to exceeding gantry and table limits at all accessible regional centers. Bedside wide local excision was performed, followed by urgent below-knee amputation for progressive necrosis. Postoperatively, the patient developed septic shock, hemothorax, respiratory failure, and dialysis-requiring acute kidney injury.

**Management and outcome:**

Despite aggressive multidisciplinary intensive care, including continuous renal replacement therapy, thoracic drainage, catecholamine support, and non-invasive ventilation, diagnostic and interventional escalation remained repeatedly limited by infrastructural constraints. Owing to refractory multi-organ failure and an infaust prognosis, treatment was de-escalated to comfort-focused care. The patient died on day 24 of admission.

**Conclusion:**

This case illustrates how extreme obesity not only predisposes to lymphangiosarcoma, but may also preclude access to basic oncologic standards such as staging and operability assessment. The case highlights a critical gap in healthcare infrastructure, where patients may be systematically excluded from standard diagnostic pathways. The absence of bariatric-capable imaging and procedural infrastructure directly contributed to a loss of curative options. Healthcare systems must urgently adapt to the rising prevalence of super-morbid obesity by ensuring size-inclusive resources, or risk systematic exclusion of an expanding vulnerable population.

## Background

Obesity has reached pandemic proportions worldwide, with its prevalence nearly tripling since 1975 [1]. While most clinical attention has focused on moderate and severe obesity, the prevalence of extreme or super-morbid obesity (BMI > 60 kg/m^2^) is steadily increasing, representing a growing subgroup of patients with unique and underrecognized healthcare needs [2].

Standard imaging equipment often has weight and size limits that patients with body mass index (BMI) > 60 may exceed [2, 3]. For instance, many computer tomography (CT) and magnetic resonance imaging (MRI) scanners have table weight limits around 200–227 kg (≈ 450–500 lbs), and MRI bores are typically about 60–70 cm in diameter [4]. Exceeding these limits may result in the patient not being able to be scanned, as the gantries and tables cannot accommodate their size [5]. Due to that timely cancer staging and intervention may become impossible, directly impacting survival. Even when scans are possible, image quality is frequently compromised. Thick adipose tissue causes increased noise, scatter, and poor penetration, leading to low contrast on images [2, 6]. Radiographs may require higher radiation doses and still yield incomplete visualization of structures. Ultrasound is similarly hampered by limited tissue penetration, and anatomic landmarks can be difficult to palpate, complicating positioning [6]. These factors often result in suboptimal imaging that can delay or impede accurate diagnosis [6].

This is particularly concerning in the context of oncological and lymphatic diseases, since small tumors or lymph nodes may be obscured by underlying adiposity and staging studies can be misleading. If critical diagnostic imaging fails, clinicians may need to rely on alternative strategies, such as symptom management, empirical therapy or intraoperative findings which is far from ideal [4]. Recent case reports have highlighted the significant impact on patient care when CT scans cannot be performed due to the patient’s weight exceeding the scanner limit forcing the team to manage without imaging evidence for weeks [4]. Surveys have found that many facilities are unaware of where to refer patients for higher-capacity scanners [4]. In summary, extreme obesity can severely restrict the availability and diagnostic value of standard imaging, posing a major obstacle to the timely diagnosis of cancer or lymphatic diseases.

### Obesity, chronic lymphedema, and cutaneous angiosarcoma (Stewart–Treves syndrome)

Lymphangiosarcoma is a rare and aggressive form of angiosarcoma, often arising in the context of chronic lymphedema, as seen in Stewart–Treves syndrome. This syndrome refers to the development of cutaneous angiosarcoma in lymphedematous tissue, traditionally following mastectomy or radiation therapy, but increasingly recognized in morbidly obese individuals [7]. Given the increasing prevalence of obesity, the incidence of obesity-related angiosarcoma is likely to rise [8]. From a pathophysiological perspective, obesity-associated lymphedema typically develops when BMI exceeds 50, due to the disruption of lymphatic drainage caused by substantial adipose tissue accumulation [8]. Stagnant lymph fluid and tissue fibrosis create a pro-inflammatory environment with hypertrophied adipocytes, macrophage infiltration and cytokine release potentially promoting localized immune dysregulation and angiogenesis, which can trigger malignancy [9]. Angiosarcoma in this setting often presents as bruised or purplish enlarging plaques on chronically swollen tissue [10]. Angiosarcomas are classified into five subtypes based on etiology and location: cutaneous angiosarcoma (60%), soft-tissue angiosarcoma (25%), lymphedema-associated angiosarcoma, radiation-induced angiosarcoma, and primary breast angiosarcoma (8%) [7].

The prognosis is poor since Stewart–Treves angiosarcomas tend to metastasize early and have a high mortality rate, which emphasizes the need for early detection [9, 10].

### Therapeutic challenges in tumor treatment for morbidly obese patients

Cancer treatment in morbidly obese patients presents significant challenges across all modalities: surgery, radiation, and systemic therapy. Surgically, extreme body habitus limits visibility and access, complicates anesthesia and positioning, and may exceed the weight limits of operating and imaging tables [2, 5]. Meta-analyses have demonstrated that obese cancer patients are at significantly increased risk for postoperative wound complications compared to their normal-weight individuals, including up to a sevenfold higher odds of wound infections and dehiscence following oncologic surgery [11]. Radiation therapy is complicated by tissue attenuation, positioning difficulties, and equipment limitations. Deep skin folds can ulcerate or become infected, and standard immobilization devices often do not accommodate patients with severe obesity. Dose accuracy may be compromised by scatter and underdosing of deep-seated tumors, requiring advanced planning or high-energy beams. These technical issues can delay care and diminish treatment efficacy [5]. Systemic therapy in obese patients poses dosing challenges. Historically, doses were capped using ideal body weight to avoid toxicity, often leading to under-treatment. However, recent guidelines by the American Society of Clinical Oncology (ASCO) address that obese patients generally tolerate full-weight-based dosing of cytotoxic chemotherapy as well as normal-weight patients [12]. Dosing should be adjusted for toxicity as needed, rather than being reduced preemptively. Obesity can alter drug distribution due to an increased volume of distribution and changes in metabolism; however, research in this area is ongoing [12]. Another therapeutic hurdle is that some clinical trials have previously excluded patients with an extreme BMI, or have not reported on them. This means that evidence of the efficacy and safety of the treatment in this group is sometimes limited [13]. Morbid obesity can make every stage of cancer treatment from surgery and imaging to chemotherapy planning more complicated. Standard tools and protocols often fall short. Tailoring therapy to this patient group remains challenging, and they often require highly individualized management plans.

### Outcomes and case reports for patients with BMI > 80–100

Patients with extreme obesity (BMI ≥ 80–100, often termed "super-super obese") are underrepresented in the literature, but existing case reports highlight significant morbidity and mortality, particularly in the setting of acute illness or complex surgery [5]. Their body size often exceeds the physical capacity of imaging and surgical equipment, limiting diagnostics and interventions. CT and MRI may be inaccessible, and operating tables may not safely support the patient’s weight, restricting procedures to bedside care [6]. Establishing intravenous access and initiating dialysis also present technical challenges. Collectively, these barriers contribute to substantially poorer clinical outcomes compared to patients with normal or moderately elevated BMI [2, 5, 11].

## Case presentation

We present the case of a 54-year-old White woman with a history of morbid obesity (weight: ~ 280 kg; BMI: 106.7 kg/m^2^). The patient had been bedbound for over three years due to her obesity and bilateral stage IV lymphoedema. She was admitted for the elective resection of a newly diagnosed cutaneous angiosarcoma on her left lower leg. The patient initially expressed a wish for an initial curative treatment approach and was admitted with the intention of undergoing oncologic resection with curative intent.

She had a significant past medical history of multiple obesity-associated comorbidities, including arterial hypertension, chronic heart failure with reduced ejection fraction (LVEF 40–45%), hypothyroidism, generalized osteoarthritis, and chronic anemia. She also had a history of pulmonary embolism, recurrent urosepsis with acute kidney injury, chronic stasis dermatitis, and multiple episodes of bilateral lower-limb erysipelas and intertrigo. Additionally, she suffered from chronic skin breakdown, multiple multidrug-resistant infections and frequent lymphorrhea.

### Clinical findings, diagnostic assessment and therapeutic intervention

The angiosarcoma had been biopsied one month prior and was located on the dorsomedial aspect of her left calf, presenting as an exulcerated, violaceous, indurated plaque with associated pain and rapid progression. Histopathology confirmed a high-grade cutaneous angiosarcoma, arising in the setting of chronic lymphostasis. The patient was admitted for oncologic resection; however, due to her extreme body size, standard diagnostic imaging for staging including CT, MRI, and PET could not be performed. The patient exceeded the weight and girth capacities of available radiological equipment, with no nearby referral options available for bariatric imaging. Ultrasound diagnostic was attempted but was technically limited and non-diagnostic due to significant overlying soft tissue, poor contrast, and scatter.

**Fig. 1:**
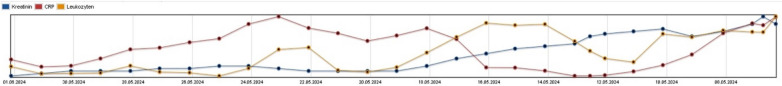
Trends in inflammatory and renal parameters during Intensive Care Unit Stay. C-reactive protein (CRP) levels (red line) indicate fluctuating systemic inflammation, with multiple peaks suggestive of recurrent septic episodes. Leukocyte count (yellow line) shows marked elevations correlating with clinical deterioration, especially around mid-May. Serum creatinine (blue line) demonstrates a progressive rise during the Intensive Care Unit course, consistent with acute kidney injury and evolving renal dysfunction, requiring continuous renal replacement therapy

Pre-operatively, the patient was already critically deconditioned and bedbound. On the peripheral ward, she developed acute anuria and signs of systemic infection, prompting emergent transfer to the intensive care unit (Fig. 1). On admission to intensive care unit (ICU), she was hypotensive, oliguric, and catecholamine-dependent. Initial labs suggested septic shock with elevated inflammatory markers, metabolic acidosis, hypoalbuminemia, and worsening renal function. Given her multiple antibiotic allergies, empiric broad-spectrum coverage with meropenem was initiated.

**Fig. 2:**
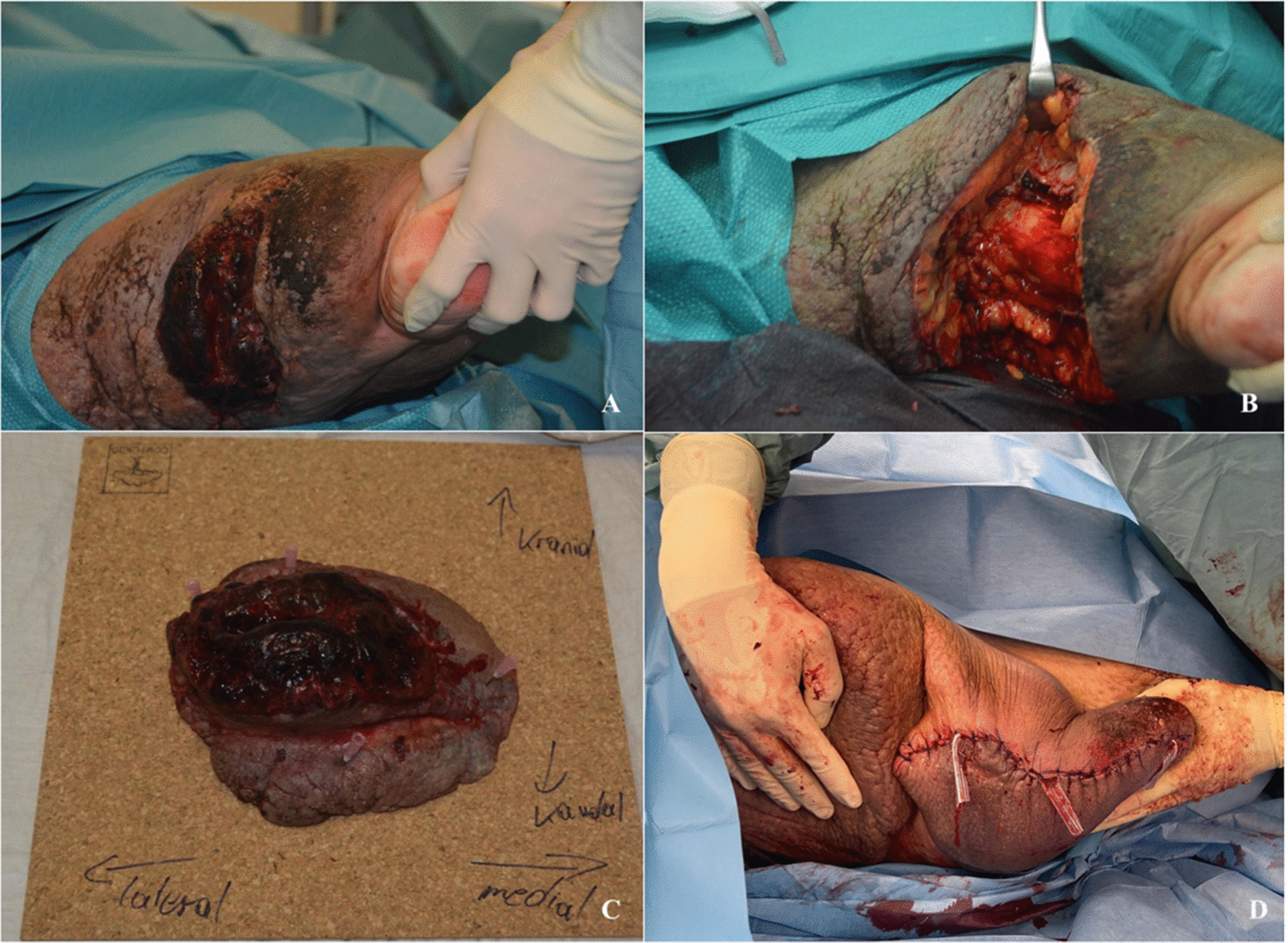
Operative management of angiosarcoma of the lower left limb in a super-morbidly obese patient sequential intraoperative photographs documenting the management of a high-grade cutaneous angiosarcoma on the lower left leg in a patient with BMI > 100. A: Preoperative view of the angiosarcoma lesion on the dorsomedial aspect of the lower left leg, showing an ulcerated, violaceous mass with surrounding lymphedematous and fibrotic skin. B: Intraoperative view immediately after wide local resection of the tumor, revealing deep infiltration into subcutaneous and muscular layers. C: Macroscopic specimen of the excised angiosarcoma with orientation markings (cranial, caudal, medial, lateral) for pathological evaluation. D: Postoperative view after below-knee amputation, performed due to progressive tissue necrosis and persistent lymphorrhea following resection

Due to her rapidly deteriorating condition and inability to undergo standard pre-operative imaging or transport, a multidisciplinary decision was made to perform a bedside wide local excision of the tumor under regional anesthesia (Fig. 2). On May 8, 2024, the left lower leg angiosarcoma was excised with oncologic intent. However, in the following days, the patient developed massive lymphorrhea from surrounding wounds, leading to significant protein loss, intravascular depletion, and worsening respiratory and renal function. Despite receiving aggressive fluid resuscitation and albumin replacement therapy, her condition deteriorated and she became increasingly hemodynamically unstable.

By hospital day 5, she developed a large, right-sided pleural effusion causing orthopnea and hypoxemia, particularly in the right lateral position. Thoracic ultrasound and bedside chest X-ray revealed a likely hemothorax, which was successfully drained via Bülau chest tube on May 13, relieving over 1.5 liters of bloody fluid. Simultaneously, the patient developed new-onset atrial fibrillation with rapid ventricular response, requiring Amiodarone perfusion and rate control. Despite this, her respiratory status remained tenuous, with progressive CO₂ retention and frequent need for non-invasive ventilation (NIV) and high-flow oxygen therapy (Table 1).

**Table 1:** Timeline of diagnosis and interventions

Timeline	
Date-hospital day	Event
06 May 2024—Day 0	Admission with biopsy-confirmed cutaneous angiosarcoma of the left lower leg with stage IV lymphedema Diagnosis of sepsis. Start of meropenem therapy. Known colonization with multiresistant Gram-negative bacteria (3-MRGN) in the urine
08 May 2024—Day 2	Bedside oncologic resection of the lymphangiosarcoma (dorsomedial left lower leg) under regional anesthesia; large open wound left due to extensive soft tissue involvement
10 May 2024–Day 4	Transesophageal echocardiography (TEE) performed to rule out endocarditis; revealed severely reduced left ventricular ejection fraction (≈40–45%)
12 May 2024—Day 6	Development of massive right pleural effusion with respiratory compromise. Initiation of continuous veno-venous hemofiltration (CVVH) due to acute kidney injury and volume overload
13 May 2024—Day 7	Right-sided hemothorax, likely spontaneous under severely impaired coagulation status. CVVH temporarily discontinued Bülau chest tube placed with evacuation of hemorrhagic fluid
16 May 2024—Day 10	Urgent bedside below-knee amputation of the left leg with primary closure due to necrotic progression and uncontrollable lymphorrhea
18 May 2024—Day 12	Development of hyperactive delirium, requiring pharmacologic management
20 May 2024—Day 14	Recurrent right-sided pleural effusion versus hemothorax suspected; repeated attempted imaging limited by body habitus
23 May 2024—Day 17	Diagnosis of pneumonia (Enterobacterales). Meropenem re-initiated (22 – 27 May)
24 May 2024—Day 18	Intermittent non-invasive ventilation (NIV) initiated due to progressive respiratory insufficiency and hypercapnia
31 May 2024—Day 25	Transition to do-not-resuscitate (DNR) / Do-Not-intubate (DNI) status after multidisciplinary and family discussions
01 June 2024—Day 26	Death due to refractory multi-organ failure despite maximal supportive therapy

On May 16, necrotic progression of the surgical site necessitated a below-knee amputation of the left leg (Fig. 2). Once again, due to logistical limitations, the procedure was performed bedside in the ICU, under high-dose catecholamine support. Postoperatively, the patient remained hemodynamically unstable, and continuous veno-venous hemofiltration (CVVH) was initiated for oliguric acute kidney injury and volume overload (Table 1). Despite transient improvements, fluid management remained precarious due to persistent lymph leakage from residual skin defects and pressure-related edema.

On May 18, the patient developed a hyperactive delirium, treated with antideliriants and intermittent dexmedetomidine. Despite ongoing respiratory and circulatory support, her condition worsened, and repeated attempts at thoracic imaging failed due to body habitus limitations.

### Outcome

Despite maximal medical management including respiratory support, antimicrobial therapy, albumin replacement, and renal support her overall condition continued to worsen. Given her infaust prognosis from angiosarcoma which was unable to be staged, her multi-organ dysfunction, and poor baseline functional reserve, multiple family discussions were held in conjunction with palliative care and ethics consultation. In agreement with her legal representative, a do-not-resuscitate (DNR) and do-not-intubate (DNI) status was established.

On May 31, the patient experienced progressive respiratory failure, worsening renal function, and escalating catecholamine needs. In consensus with the treating team and her guardian, a decision was made to discontinue intensive life-sustaining interventions. She passed away peacefully on June 1, 2024, 26 days after admission (Table 1).

## Discussion

The worldwide prevalence of obesity has nearly tripled between 1975 and 2016 and has now reached pandemic dimensions [14]. The prevalence of the rising prevalence of obesity particularly at its most extreme levels poses a growing challenge not only for individual patient care but for the structural and logistical capacities of modern healthcare systems. Morbid obesity (BMI > 40 kg/m^2^), and especially super-morbid obesity (BMI > 60–80 kg/m^2^), is no longer rare. With global obesity rates climbing steadily, hospitals are increasingly encountering patients whose body habitus exceeds the limits of conventional diagnostics, therapeutics, and infrastructure.

From a systemic standpoint, extreme obesity strains nearly every facet of acute and chronic medical care. As mentioned before imaging modalities such as CT and MRI and surgical tables, transport equipment and radiotherapy machines often have weight and bore-size restrictions, which can delay or prevent accurate diagnosis and treatment [5, 6]. While some high-capacity CT scanners can accommodate weights up to approximately 300–350 kg, such systems remain scarce and are typically limited to specialized centers. MRI systems are further restricted by bore diameter, which often prevents scanning even when weight limits are not exceeded [15].

These infrastructural limitations are compounded by clinical challenges: physical examination is more difficult, venous access may be compromised, and anesthesia carries increased risk due to altered physiology and comorbid conditions. Such barriers result in diagnostic delays, incomplete staging in oncologic care, and, in some cases, the inability to perform lifesaving procedures.

The case presented here underscores the devastating consequences when standard healthcare workflows are not designed to serve the full spectrum of body sizes. This case should therefore be understood not only as a report of angiosarcoma in a high-risk patient, but primarily as an illustration of how infrastructural limitations can directly prevent the delivery of standard oncologic care.

This patient’s extreme obesity not only contributed to the development of chronic lymphedema a known precursor for cutaneous angiosarcoma but also prevented timely imaging, comprehensive staging, and safe access to curative treatment options. These realities culminated in a clinical scenario defined by therapeutic limitations and a poor outcome, despite multidisciplinary efforts.

Furthermore, obesity is strongly associated with an increased risk of numerous chronic and acute diseases, including type 2 diabetes, cardiovascular disease, obstructive sleep apnea, renal impairment, and several forms of cancer [16]. Obesity-related immune dysregulation, impaired wound healing, and chronic inflammation all contribute to higher complication rates and poorer prognoses across multiple medical domains [16, 17].

It is predicted that obesity will become the leading preventable risk factor for non-communicable diseases (NCDs) by 2035 [18]. As such, there is an urgent need for robust and sustained public health strategies aimed at prevention. This includes promoting nutritional education, increasing access to physical activity, addressing social determinants of health, and implementing early screening and intervention programs particularly in children and adolescents, where long-term impact can be greatest. Preventing the progression of obesity is not only a medical imperative but also a socioeconomic one: reducing obesity prevalence will directly reduce the incidence and severity of NCDs and lessen the burden on overstretched healthcare systems.

Healthcare institutions must adapt in parallel. Proactive infrastructure planning, investment in bariatric-capable equipment, development of obesity-sensitive clinical protocols, and multidisciplinary training are now essential. Without such changes, the health system risks reinforcing disparities and denying equitable care to a growing and vulnerable patient population.

## Conclusion

The increasing prevalence of obesity, and particularly super-morbid obesity, has serious implications for patients, healthcare providers and entire healthcare systems. Beyond posing individual health risks, obesity challenges the design and delivery of medical care itself. Consequences for patients who exceed the functional limits of standard equipment and care pathways include diagnostic delays, limited treatment options and higher procedural risk. Our case study illustrates the cumulative risks associated with obesity-related diseases, especially when exacerbated by chronic conditions such as lymphedema and aggressive malignancies like angiosarcoma. Ultimately, this case highlights that extreme obesity is not only a medical condition but also a system-level challenge, requiring adaptation of healthcare infrastructure to ensure equitable access to diagnosis and treatment. As rates of severe obesity continue to rise, healthcare systems must respond with proactive infrastructure planning, bariatric-specific care protocols, and continued emphasis on early prevention, patient-centered management, and equitable access to care. Public health strategies that target the roots of obesity are essential to reverse current trends. Early intervention, education, and health promotion efforts will be key to reducing the future burden of obesity-related diseases.

Ultimately, a healthcare system that aims to serve every patient must evolve to meet the needs of all regardless of size, complexity, or circumstance.

## Data Availability

The datasets used and/or analyzed during the current study are available from the corresponding author on reasonable request.

## Abbreviations

ASCO: American Society of Clinical Oncology
BMI: Body mass index
CT: Computer tomography
CVVH: Continuous veno-venous hemofiltration
DNI: Do-not-intubate
DNR: Do-not-resuscitate
ICU: Intensive Care Unit
MRI: Magnetic resonance imaging
NCD: Non-communicable diseases
NIV: Non-invasive ventilation
